# Reclassification of breast cancer: Towards improved diagnosis and outcome

**DOI:** 10.1371/journal.pone.0217036

**Published:** 2019-05-22

**Authors:** Alexander P. Landry, Zsolt Zador, Rashida Haq, Michael D. Cusimano

**Affiliations:** 1 Department of Surgery, St. Michael’s Hospital, Toronto, ON, Canada; 2 Faculty of Medicine, University of Toronto, Toronto, ON, Canada; 3 Division of Hematology/Oncology, St. Michael’s Hospital, Toronto, ON, Canada; 4 Dalla Lana School of Public Health, University of Toronto, Toronto, ON, Canada; University of South Alabama Mitchell Cancer Institute, UNITED STATES

## Abstract

**Background:**

The subtyping of breast cancer based on features of tumour biology such as hormonal receptor and HER2 status has led to increasingly patient-specific treatment and thus improved outcomes. However, such subgroups may not be sufficiently informed to best predict outcome and/or treatment response. The incorporation of multi-modal data may identify unexpected and actionable subgroups to enhance disease understanding and improve outcomes.

**Methods:**

This retrospective cross-sectional study used the cancer registry Surveillance, Epidemiology and End Results (SEER), which represents 28% of the U.S. population. We included adult female patients diagnosed with breast cancer in 2010. Latent class analysis (LCA), a data-driven technique, was used to identify clinically homogeneous subgroups (“endophenotypes”) of breast cancer from receptor status (hormonal receptor and HER2), clinical, and demographic data and each subgroup was explored using Bayesian networks.

**Results:**

Included were 44,346 patients, 1257 (3%) of whom had distant organ metastases at diagnosis. Four endophenotypes were identified with LCA: 1) “*Favourable biology”* had entirely local disease with favourable biology, 2) “*HGHR-”* had the highest incidence of HR- receptor status and highest grade but few metastases and relatively good outcomes, 3) “*HR+ bone”* had isolated bone metastases and uniform receptor status (HR+/HER2-), and 4) “*Distant organ spread”* had high metastatic burden and poor survival. Bayesian networks revealed clinically intuitive interactions between patient and disease features.

**Conclusions:**

We have identified four distinct subgroups of breast cancer using LCA, including one unexpected group with good outcomes despite having the highest average histologic grade and rate of HR- tumours. Deeper understanding of subgroup characteristics can allow us to 1) identify actionable group properties relating to disease biology and patient features and 2) develop group-specific diagnostics and treatments.

## Background

Significant research efforts have been undertaken to identify markers of breast cancer biology and prognosis. Gene expression profiling has elucidated five “intrinsic” subgroups of the disease: *luminal A*, *luminal B*, *HER2-overexpressing*, *basal-like*, and *normal-like*[1,2]. Conveniently, hormone receptors (HR; estrogen and progesterone) and human epidermal growth factor receptor 2 (HER2) function as classification biomarkers; their status can approximate intrinsic subtypes without necessitating cumbersome genetic profiles (*luminal* and *normal-like* are HR+, *HER2-overexpressing* is HR-/HER2+, and *basal-like* is HR-/HER2-)[3,4]. The HR-/HER2- subtype, also called “triple-negative” (TN) disease since it lacks all three receptors, is associated with the worst prognosis[2,5]. Importantly, the molecular classification of breast cancer has led to the development of targeted therapies which have improved survival in primary disease[6]. However, while HER2-targeted treatments also show promise in the setting of metastasis[7], outcomes in metastatic disease have not improved significantly over the last several years[7,8] and only about 30% of stage 4 breast cancers are HER2+[4] and thus amenable to this therapy. Further, the effectiveness of HER2 treatment in the setting of brain metastasis is limited by blood brain barrier (BBB) impermeability[9]. Novel classification systems have the potential to improve our understanding of metastatic breast cancer and further inform the clinical management of this challenging disease state.

The clinical course and prognosis of breast cancer are highly variable[10,11]. Identifying mutually exclusive subgroups of patients within a heterogeneous patient population is called endophenotype discovery[12–14], and has translated into practice-changing recommendations in the management of asthma[12], ARDS[13] and sepsis[14]. Latent class analysis (LCA) has been used for this purpose in the clinical setting[13,15,16], and is preferred over conventional clustering[17]. This technique is based on the assumption that there exists an unobserved categorical variable, inferable from a set of observed variables, which groups subjects into “latent” classes based on individual similarities[18]. The technique of endophenotype discovery can ultimately inform subgroup-specific treatment plans (e.g. identifying tumour-specific drugs), trial design (e.g. testing prophylactic irradiation strategies for high risk groups) and prognostication. Bayesian Network Analysis (BNA), a machine learning based method for computing and visualizing probabilistic associations within a dataset[19], is useful for further data exploration and hypothesis generation, and has been applied to hepatocellular carcinoma[20], lung cancer[21], traumatic brain injury[19], and subarachnoid hemorrhage[22]. Network output is visualized with a directed acyclic graph (DAG), wherein probabilistic associations between nodes (parameters) are indicated by edges (arrows). The utility of these novel approaches in breast cancer has not been adequately explored, and it may provide further insight into this heterogeneous disease.

The purpose of this work is to identify novel subgroups of breast cancer using a large cancer repository, and explore groups for whom current classification systems are inadequate. This will serve as an important guide for future work aimed at constructing deeper, more informative endophenotypes of this disease.

## Methods

### Data collection

Data was extracted from the 2016 edition of the open-source cancer registry Surveillance, Epidemiology and End Results (SEER). SEER encodes data on individual cancer cases in the USA from 18 population-based registries and represents approximately 28% of the country’s population[23]. Sites of distant metastases and hormone receptor/HER2 statuses of primary breast tumours have been recorded since 2010. Consequently, we limited our study to cases diagnosed in 2010 to maximize follow up and minimize time-related cohort heterogeneity. Demographic information for each case included age (<50 vs. 50–69 vs. ≥70 years old)[4], race (white vs. black vs. other), marital status (married vs. unmarried) and health insurance status (insured vs. uninsured). Disease biology at diagnosis was described by estrogen and progesterone receptor status (present vs. absent vs. borderline), HER2 status (present vs. absent), and grade (1–4) of the primary tumour. Primary tumour size (<2 cm vs. 2–4.9 cm vs. ≥5 cm)[4] as well as lymph node and distant organ (brain, liver, lung, bone) metastases (present vs. absent) served to describe disease burden at the time of diagnosis. Finally, we assessed overall survival based on the most recent documented follow-up (dead vs. alive). Following a recent SEER-based study from Gong et al[4], estrogen receptor (ER) and progesterone receptor (PR) status coded as “borderline” in the database were defined as positive in our study.

### Inclusion/Exclusion criteria

This study included female patients from the 2016 SEER registry who were diagnosed with breast cancer during the year 2010. All included patients were at least 18 years old at the time of diagnosis. We required cases to have been diagnosed by histology and excluded those with a diagnosis made at autopsy or based on a death certificate alone[4]. We also excluded cases if any included parameters (defined in the previous subsection) were coded as “unknown” in the database. Similarly, cases with “borderline” HER2 status were not included in the analysis[4].

### Endophenotype discovery and hypothesis generation

Comparison was first made between patients with and without distant organ metastases at diagnosis using contingency tables for each parameter and applying a chi-squared test of independence. Building and testing an LCA model then served as the primary tool for endophenotype discovery. Since these models are best suited for binary classification variables, we used the status of receptors (HR, HER2) and metastases (lymph nodes, brain, lung, liver, and bone) for this purpose. The optimal number of classes was determined by computing the Bayesian Information Criterion (BIC) for models with between 2 and 7 classes and selecting the configuration which minimized the BIC. Contingency tables were generated for each parameter and a chi-square test used to probe inter-class differences. For descriptive purposes, the LCA was repeated 10 times to observe the degree of reproducibility.

Bayesian networks were used to explore variable interactions within each endophenotype and thus further probe the characteristics of each class. A hill-climbing algorithm was used to train the networks[19], and BIC values were used to represent association strengths (with an inverse proportionality). In the DAG representation, arrows with tails at the “survival” node were omitted, as a distal outcome cannot logically influence a patient’s state at diagnosis. Results from the BNA were validated using structural equation modelling (SEM), a statistical tool capable of assessing the fit of a theoretical model on a dataset using inter-related multiple regression models. Root mean squared error of approximation (RMSEA) and standardized root mean squared residual (SRMR), both common measures of absolute model fit (wherein the former includes a penalty for model complexity and the latter does not) were used to assess the Bayesian model configurations. We consider fits to be adequate when both RMSEA and SRMR are < 0.05 (based on recommended cutoffs of 0.06 and 0.08, respectively[24]).

Notably, both LCA and BNA were used to explore potential metastatic “drivers” (factors which predispose to metastasis) and “organ-selectors” (factors which influence the location of a metastasis, without necessarily affecting the probability of one actually occurring). A p-value ≤ 0.001 was considered significant in this study.

### Computational platform

All analysis and modelling used R, an open-source platform for statistical computation and graphics[25]. The packages “poLCA”[26], “bnlearn”[27], and “lavaan”[28] were used for LCA, BNA, and SEM, respectively.

## Results

### Patient characteristics

An overview of patient characteristics is presented in Table 1. The majority are white (81.1%), insured (98.4%) and married (72.1%). Most primary tumours are <2 cm (57.2%) and HR+/HER2- (73.3%). About one third of patients have lymph node metastases at diagnosis (32%) but distant organ metastases are rare, with bone being the most common site (2.1%) and brain being the least common (0.2%). Most patients are alive at the most recent documented follow up (86.3%). In the comparison between those with and without organ metastases at diagnosis, all parameters except age and marital status are significantly different (p<0.001, chi square). Patients presenting with metastasis are more likely to be black, uninsured, and unmarried but these differences are small in absolute terms. The metastasis group also has larger and higher grade primary tumours which are more likely to be HR-, and be associated with greater lymph node burden and comparatively poor survival.

**Table 1:** Demographics of the final dataset.

N (%)	All cases	No metastases	Metastases
	44346 (100)	43089 (97.2)	1257 (2.8)
**Age**			
<50	9491 (21.4)	9204 (21.4)	287 (22.8)
50–69	22365 (50.4)	21713 (50.4)	652 (51.9)
≥70	12490 (28.2)	12172 (28.2)	318 (25.3)
**Race** *			
White	35959 (81.1)	34992 (81.2)	967 (76.9)
Black	4642 (10.5)	4433 (10.3)	209 (16.6)
Other	3745 (8.4)	3664 (8.5)	81 (6.4)
**Insurance** *			
Uninsured	722 (1.6)	678 (1.6)	44 (3.5)
Insured	43624 (98.4)	42411 (98.4)	1213 (96.5)
**Marital status**			
Unmarried	12385 (27.9)	11999 (27.8)	386 (30.7)
Married	31961 (72.1)	31090 (72.2)	871 (69.3)
**Grade** *			
1	10236 (23.1)	10126 (23.5)	110 (8.8)
2	19376 (43.7)	18848 (43.7)	528 (42.0)
3	14556 (32.8)	13952 (32.4)	604 (48.1)
4	178 (0.4)	163 (0.4)	15 (1.2)
**Size** *			
<2cm	25360 (57.2)	25170 (58.4)	190 (15.1)
2–4.99cm	15277 (34.4)	14698 (34.1)	579 (46.1)
≥5 cm	3709 (8.4)	3221 (7.5)	488 (38.8)
**Receptor Subtype** *			
HR+/HER2+	4438 (10.0)	4264 (9.9)	174 (13.8)
HR+/HER2-	32526 (73.3)	31748 (73.7)	778 (61.9)
HR-/HER2+	1948 (4.4)	1838 (4.3)	110 (8.8)
HR-/HER2-	5434 (12.3)	5239 (12.2)	195 (15.5)
**Nodes** *			
Negative	30137 (68.0)	29852 (69.3)	285 (22.7)
Positive	14209 (32.0)	13237 (30.7)	972 (77.3)
**Metastases** †			
Brain	98 (0.2)	0 (0)	98 (7.8)
Liver	346 (0.8)	0 (0)	346 (27.5)
Lung	396 (0.9)	0 (0)	396 (31.5)
Bone	914 (2.1)	0 (0)	914 (72.7)
**Survival** *			
Dead	6095 (13.8)	5249 (12.2)	846 (67.3)
Alive	38251 (86.3)	37840 (87.8)	411 (32.7)

* p < 0.001

† p value not applicable

### Endophenotype discovery with latent class analysis

**Fig 1:**
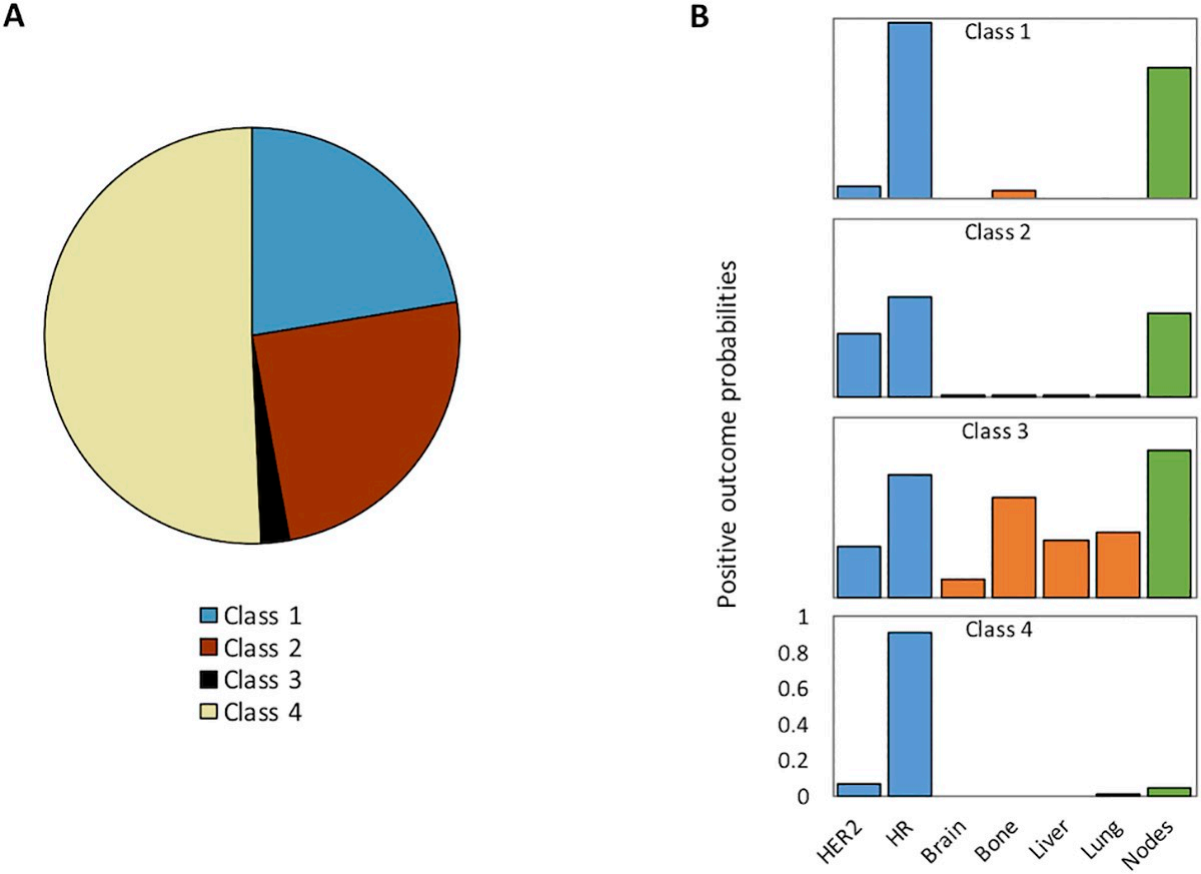
LCA model output. A: Probabilistic distribution of patients by latent class. Note that this represents the probability of a patient being classified in each subgroup, and is not equivalent to the distribution of cases in this study (Table 3). B: Outcome probabilities for each class. Each bar represents the probability of a parameter being positive in a particular class. Bars are colour coded based on the following groupings: HR/HER2 subtype (blue), distant organ metastases (orange), and lymph node metastases (green).

A four-latent-class model was selected based on BIC values, which demonstrate good reproducibility (Table 2). LCA outcome probabilities are shown in Fig 1, and a frequency table of class compositions is presented in Table 3. We observe unambiguous HR/HER2 statuses in classes 1 and 4 (high likelihood of HR+/HER2-) yet greater heterogeneity in classes 2 and 3, a high probability of metastases in group 3, and varying probabilities of lymph node metastasis between groups (Fig 1). In terms of case distribution (Table 3), class 4 accounts for the majority of cases, and is associated with the best survival. Patients are more likely to be older and white and none have lymph node or distant organ metastases at diagnosis. This group also has the smallest, lowest grade primary tumours which are mostly HR+/HER2-. Class 2 is the youngest, with higher grade tumours which are more likely to be HR-/HER2+ or TN. Despite higher grade and HR/HER2 status, tumour size and lymph node involvement are moderate, there are very few distant organ metastases (none to bone; no patients have metastases to more than one site), and survival is quite good. Class 1, which is entirely HR+/HER2-, has an especially high lymph node burden and contains approximately half the total number of bone metastases (without any metastases to other sites). Class 3 has the worst prognosis and accounts for the vast majority of metastatic burden at diagnosis (all patients have distant organ involvement); patients are also more likely to have larger primary tumours and to be black, uninsured, and unmarried. Interestingly, this group has fewer lymph node metastases than class 1 and tumours are lower grade and are less likely to be HR-/HER2+ or TN compared to class 2. Based on these observations, we will refer to these endophenotypes as follows: *favourable biology* (class 4), *HGHR-* (class 2), *HR+ bone* (class 1), and *distant organ spread* (class 3). In Table 3, there are highly significant differences (p<0.001, chi square) between classes for all parameters. Notably, similar LCA outputs were observed in 9/10 repetitions.

**Table 2:** LCA model optimization. Note that BIC is minimized with 4 classes.

Classes	BIC	BIC (repeated)
2	147101.6	147102.6
3	146085.0	146082.0
**4**	**146019.7**	**146055.0**
5	146145.4	146110.4
6	146175.5	146213.9
7	146273.1	146266.7

**Table 3:** Latent class compositions.

N (%)	Class 1	Class 2	Class 3	Class 4
	9585 (21.6)	5187 (11.7)	770 (1.7)	28804 (65.0)
**Age** *				
<50	2500 (26.1)	1610 (31.0)	177 (23.0)	5204 (18.1)
50–69	4914 (51.3)	2586 (49.9)	408 (53.0)	14457 (50.2)
≥70	2171 (22.6)	991 (19.1)	185 (24.0)	9143 (31.7)
**Race** *				
White	7799 (81.4)	3846 (74.2)	35959 (72.5)	23756 (82.5)
Black	1050 (11.0)	822 (15.8)	4642 (19.7)	2618 (9.1)
Other	736 (7.7)	519 (10.0)	3745 (7.8)	2430 (8.4)
**Insurance** *				
Uninsured	189 (2.0)	144 (2.8)	30 (3.9)	359 (1.2)
Insured	9396 (98.0)	5043 (97.22)	740 (96.1)	28445 (98.8)
**Marital status** *				
Unmarried	2513 (26.2)	1386 (26.7)	251 (32.6)	8236 (28.6)
Married	7072 (73.8)	3802 (73. 3)	519 (67.4)	20568 (71.4)
**Grade** *				
1	1703 (17.8)	119 (2.3)	42 (5.5)	8372 (29.1)
2	5023 (52.4)	1266 (24.4)	268 (34.8)	12819 (44.5)
3	2821 (29.4)	3755 (72.4)	448 (58.2)	7532 (26.1)
4	38 (0.4)	47 (0.9)	12 (1.6)	81 (0.3)
**Size** *				
<2cm	3438 (35.9)	1713 (33.0)	118 (15.3)	20091 (69.8)
2–4.99cm	4625 (48.3)	2544 (49.0)	339 (44.0)	7769 (27.0)
≥5 cm	1522 (15.9)	930 (17.9)	313 (40.6)	944 (3.3)
**Receptor Subtype** *				
HR+/HER2+	0 (0)	1609 (31.0)	163 (21.2)	2666 (9.3)
HR+/HER2-	9585 (100)	14 (0.3)	320 (41.6)	22607 (78.5)
HR-/HER2+	0 (0)	1844 (35.6)	104 (13.5)	0 (0)
HR-/HER2-	0 (0)	1720 (33.2)	183 (23.8)	3531 (12.3)
**Nodes** *				
Negative	112 (1.2)	1091 (21.0)	130 (16.9)	28804 (100)
Positive	9473 (98.8)	4096 (79.0)	640 (83.1)	0 (0)
**Metastases** *				
Brain	0 (0)	1 (0.02)	97 (12.6)	0 (0)
Liver	0 (0)	28 (0.5)	318 (41.3)	0 (0)
Lung	0 (0)	14 (0.3)	382 (49.6)	0 (0)
Bone	444 (4.6)	0 (0)	470 (61.0)	0 (0)
Any site	444 (4.6)	43 (0.8)	770 (100)	0 (0)
**Survival** *				
Dead	1574 (16.4)	1180 (22.7)	555 (72.1)	2786 (9.7)
Alive	8011 (83.6)	4007 (77.3)	215 (27.9)	26018 (90.3)

* p < 0.001

### Bayesian network analysis

**Fig 2:**
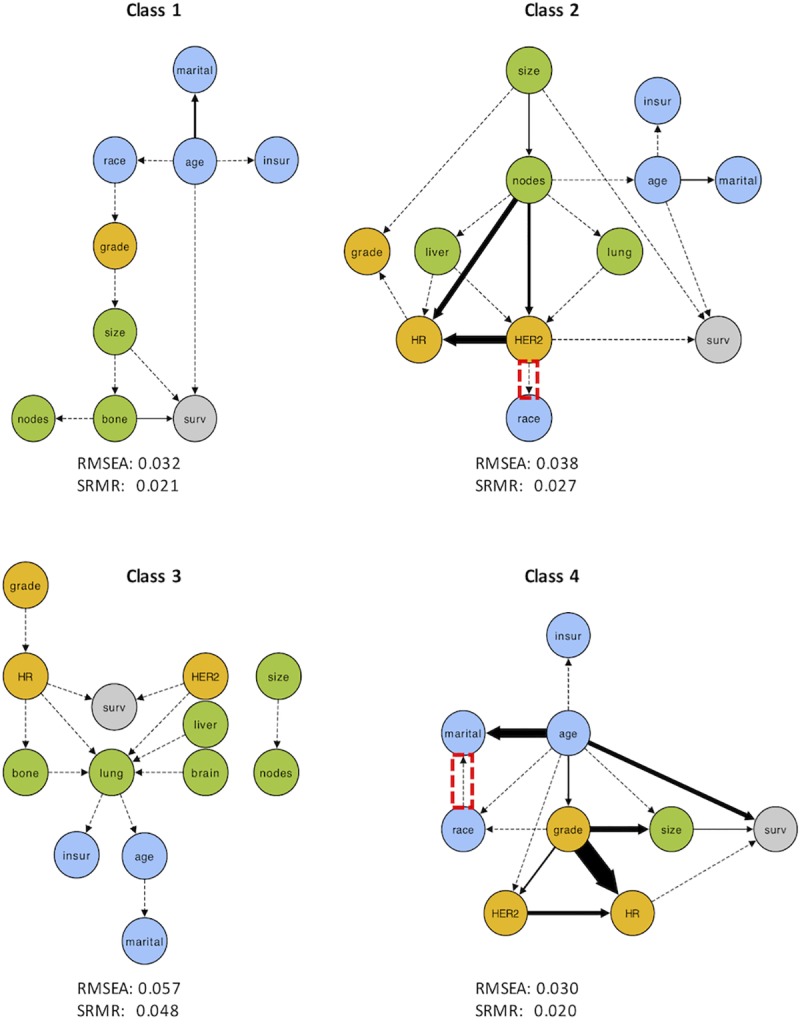
DAG plots for each latent class. Arrow thickness represents the strength of association, which is inversely proportional to BIC. Dotted lines represent associations which are ≤5% of the strength of the strongest association. Node colours are grouped as follows: host features (blue), tumour biology (orange), disease burden (green), and overall survival (grey). Red boxes indicate interactions which were not validated by the SEM regression (p > 0.001). RMSEA = root mean square error of approximation; SRMR = standardized root mean square residual; “surv” = survival; “insur” = insurance.

We present the DAG outputs of the BNA in Fig 2. In class 1, age and race are proximal features which are associated with disease-specific parameters (grade, spread) and survival. Class 2 is notable for having tumour size and spread (nodes, liver, lung) as proximal variables influencing HR/HER2 subtype, demographic features and survival. Age and HER2 status are directly associated with survival, though the hormone receptor status has no significant effect. In class 3, demographic features don’t appear to have a significant effect on disease or survival despite the increased proportion of patients who are black, uninsured, and unmarried. Receptor subtype affects survival and HR status influences site of metastasis (bone/lung), though there is no significant association between site of metastasis and mortality. Finally, age directly influences all variables (except HR status) in class 4, and survival is also a direct function of size and HR status.

### Structural equation modelling

A single discrepancy is noted between BNA and SEM in classes 2 and 4 (Fig 2). However, each Bayesian model achieved an adequate fit (with the discrepant associations removed) based on the RMSEA and SRMR values, indicating they are plausible constructs with which to describe this data.

## Discussion

### Overview of key results

Latent class analysis identified four endophenotypes of breast cancer: *favourable biology*, with biologically favourable and locally contained tumours; *HGHR- (“high grade HR-“)*, with the highest grade tumours on average and largely HR- receptor subtypes, yet limited disease spread and good survival; *HR+ bone*, described by HR+/HER2- subtype, lymph node and isolated lung metastases, and good survival; *distant organ spread*, which represents the vast majority of metastatic burden and death despite less aggressive tumour grade and HR/HER2 status than the *HGHR-* cohort. Bayesian networks identified differences in variable interactions among classes as well.

### The “HGHR-” class has unexpected features

The *HGHR-* group is of particular importance due to its unexpected features. As the cohort with the highest grade tumours which are predominantly HR-/HER2+ and TN, its histopathology would classically be considered unfavourable. It is therefore surprising that this is not the group with the highest metastatic burden and lowest survival. It seems that while HR/HER2 status may be an “organ-selector” of metastasis it may not be a key “driver” of them; i.e. cannot be used to reliably predict whether or not a metastasis will occur in the first place. This postulate is based on the fact that this endophenotype has so few metastases and that HR status does not significantly drive metastases in this cohort’s BNA. Alternatively, these patients may still develop metastases with time but have prolonged survival nevertheless.

The relationship between hormone receptor and HER2 subtype and preferential site of metastasis is not new, and it is known that HR+ tumours are associated with spread to bone while HR- tumours primarily distribute to liver, lung, or brain[4]. This is corroborated by observing differences between *HR+ bone* (HR+ only, bone is the only site of metastasis) and *HGHR-* (mostly HR-, no bone metastases) and by noting the influence of HR status on site of metastasis in the *distant organ spread* BNA (Fig 2; since all patients have metastases, influence is purely on location). Nevertheless, there is considerable overlap in sites of tumour spread[4] and predictions are largely limited to “bone vs. non-bone”. These observations indicate that HR/HER2 subtype alone is not a sensitive nor specific predictor of metastasis, and clearly suggest a need for further exploration of prediction models.

### Social factors may play a role in metastasis and outcome

There appears to be a relationship between socioeconomic status and disease burden/outcome as observed in the *distant organ spread* endophenotype (Table 3). Additionally, race directly influences tumour grade in the *HR+ bone* BNA (Fig 2) and the relationship between race and HR (which, in turn, is related to tumour grade) has been suggested to be mediated by socioeconomic factors rather than genetics alone[29]. The relevance of host features is not surprising as it has previously been shown that indicators of socioeconomic status including age, marital status, and occupational status are predictors of breast cancer mortality[4,30,31] and stage at diagnosis[30]. This may be mediated by a lack of access to healthcare, differences in exposures/behaviours, and/or underlying biology. For example, better access to care may have played a role in the *HGHR-* group’s favourable outcomes. Addressing barriers of care will be critical in this subset of patients in order to prevent early disease spread and consequent poor outcomes.

### Limitations

Several limitations of this study must be considered. This study is cross sectional in nature. SEER does not record data beyond the time of diagnosis, making it impossible to track disease evolution (i.e. the development of new metastases with time). This hindered our ability to assess metastatic risk based on a patient’s state at diagnosis. The nature of the dataset, with the rarity of distant organ metastases at diagnosis, may also have limited the statistical power of our results, particularly in the BNA. This limited our abilities to draw conclusions, particularly relating to brain and liver metastases which are associated with particularly dismal outcomes and are therefore very important. In addition, molecular classification available in SEER was based on HER2 and HR status alone, which is not fully concordant with gene expression-derived intrinsic subtypes[2]. Similarly, we were not able to include potentially important parameters such as gene expression data and more extensive host information due to limited data availability. This also precluded the analysis of metastasis biology, allowing the possibility of receptor conversion from the primary tumour[9,32]. Finally, despite its size, SEER may not be fully representative of the US breast cancer population and does not consider populations outside the US. Nevertheless, the purpose of this work was to generate hypotheses using data covering significant segments of the population in order to guide the construction of deeper endophenotypes using other, more complete data sources.

### Future directions

Validation of our results by independently detecting similar subgroups based on prospectively gathered clinical, demographic, and molecular data will be an important next step. Subsequently, deriving endophenotypes of breast cancer from comprehensive gene expression profiles, in tandem with richer host phenotypes (i.e. patient characteristics), may lead to more holistic and informative classification models. Inclusion of all previously identified genes of significance in these models should be attempted in order to ensure completeness. Consequent identification of the precise phenotypes which are associated with metastases on a longitudinal basis may improve our understanding of this disease state, allow for better prevention and screening, and ultimately lead to more effective treatments.

## Conclusions

Data-driven techniques using receptor (HR and HER2) subtypes, host factors and metastatic potential from the SEER dataset identified novel unexpected endophenotypes of breast cancer and suggest the need for better classification using deeper models. Future work to expand on the endophenotypes we have described using more complete data and longitudinal designs will lead to a better ability to understand, predict, and treat metastases and ultimately improve outcomes in these patients.
